# The serial mediating role of self-esteem and health literacy in the relationship between self-efficacy and benefit finding among Chinese systemic lupus erythematosus patients

**DOI:** 10.3389/fpsyg.2026.1784999

**Published:** 2026-03-16

**Authors:** Xu Tong, Mingling Zhu, Yinsong Xu, Cancan Sun, Xuan Wang

**Affiliations:** 1 Department of Nursing, Bozhou College of Traditional Chinese Medicine, Bozhou, Anhui, China; 2 Department of Nursing, Zhejiang Shuren University, Hangzhou, Zhejiang, China

**Keywords:** benefit finding, health literacy, mediating effect, self-efficacy, self-esteem, systemic lupus erythematosus

## Abstract

**Objective:**

To investigate the serial mediating roles of self-esteem and health literacy in the relationship between self-efficacy and benefit finding among patients with systemic lupus erythematosus (SLE), and to provide empirical evidence for developing targeted clinical interventions.

**Methods:**

A cross-sectional study was conducted using convenience sampling. A total of 207 patients with SLE were recruited from the rheumatology outpatient department of a tertiary Grade-A hospital in Zhejiang Province. Data were collected using the General Information Questionnaire, the Rosenberg Self-Esteem Scale (RSES), the Self-Efficacy for Managing Chronic Disease Scale, the Health Literacy Scale, and the Benefit Finding Scale (BFS). Pearson correlation analysis and Hayes' Process macro, were used to analyze the data.

**Results:**

The mean scores for self-efficacy, self-esteem, health literacy, and benefit finding were 21.47 ± 5.37, 27.42 ± 2.68, 94.29 ± 10.71, and 65.20 ± 10.92, respectively. Correlation analysis revealed significant positive associations among self-efficacy, self-esteem, health literacy, and benefit finding (*r* = 0.833, 0.885, *P* < 0.001). Path analysis indicated that self-efficacy had a significant direct effect on benefit finding (β = 0.339, 95% CI: 0.041–0.636). The mediating effects of self-esteem (effect = 0.636, 95% CI: 0.234–1.067) and health literacy (effect = 0.506, 95% CI: 0.200–0.819) were also significant. Furthermore, the serial mediating pathway of “self-efficacy → self-esteem → health literacy → benefit finding” was significant (effect = 0.212, 95% CI: 0.042–0.577). The moderation analysis indicated that the indirect effect of self-efficacy on benefit finding via the chain mediation of self-esteem and health literacy was stronger in younger patients (β = −0.080, 95% CI: −0.120 to −0.039).

**Conclusion:**

Self-efficacy is positively associated with benefit finding in patients with SLE directly and indirectly through self-esteem and health literacy. Age moderates the relationship between self-efficacy and self-esteem. Clinical nursing staff should adopt age-specific interventions to improve patients' self-esteem and health literacy, which may help promote their benefit finding.

## 1 Introduction

Systemic Lupus Erythematosus (SLE) constitutes a critical global public health challenge, posing severe threats not only to physical integrity but also to psychological resilience. As a chronic autoimmune disease persisting throughout the lifespan, SLE is characterized by the immune system's erroneous attack on healthy tissues, resulting in widespread inflammation across multiple organs, including the kidneys, skin, and joints (Rees et al., 2017; Durcan et al., 2019; Tsokos, 2011). The clinical course of the disease is highly unpredictable, manifesting in a complex pattern of alternating exacerbations and remissions (Ameer et al., 2022; Kaul et al., 2016). Epidemiological data indicate a high prevalence of SLE in China, with the incidence rate escalating annually (Li et al., 2013; Tian et al., 2023). Compared to the general population, patients with SLE face a significantly elevated risk of all-cause mortality and a marked reduction in survival quality (Lee Y. H. et al., 2016; Bernatsky et al., 2006). The burden imposed by SLE extends beyond physical impairment to deeply infiltrate the psychological domain. Patients endure chronic pain and debilitating fatigue, harbor persistent concerns regarding body image due to mucocutaneous manifestations, and suffer anxiety related to the adverse effects of long-term pharmacotherapy (Jolly, 2005; McElhone et al., 2006; Waldheim et al., 2013). Studies have consistently reported a high prevalence of anxiety and depression within this population (Zhang et al., 2017; Figueiredo-Braga et al., 2018). If left unaddressed, these negative emotional states can trigger heightened inflammatory responses, thereby creating a vicious cycle where psychological distress exacerbates physiological deterioration (Straub and Schradin, 2016; Rosenkranz and Davidson, 2009). Consequently, it is imperative for clinicians to transcend the traditional biomedical model and adopt a biopsychosocial approach to patient care (Wade and Halligan, 2017; Papadimitriou, 2017).

In recent years, the paradigm of psychological intervention for chronic illness has shifted from a purely pathocentric focus on alleviating depression to a salutogenic approach emphasizing the cultivation of psychological resilience. Within the framework of positive psychology, “benefit finding” has emerged as a pivotal concept, referring to an individual's capacity to perceive positive changes and meaning arising from traumatic life events or serious illness (Seligman and Csikszentmihalyi, 2000; Helgeson et al., 2006). In the struggle against disease, patients may report strengthened family cohesion, enhanced personal resilience, and a reprioritization of life values (Pakenham, 2005; Mohr et al., 1999). This positive adaptation is not merely subjective; it exerts tangible health effects. Evidence suggests that high levels of benefit finding facilitate adaptation to stress, attenuate cortisol secretion, significantly improve quality of life, and enhance treatment adherence (Antoni et al., 2001; Carver and Antoni, 2004). However, benefit finding is not an automatic outcome; significant individual variability exists. Elucidating the psychological mechanisms driving this positive adaptation is therefore essential for developing precision intervention strategies (Meng et al., 2024).

When exploring the antecedents of positive adaptation, self-efficacy is postulated as a primary determinant of behavioral change. Rooted in Bandura's Social Cognitive Theory, self-efficacy denotes an individual's belief in their capability to execute necessary actions in specific situations (Bandura, 1997, 2004). In the context of chronic disease management, it specifically refers to a patient's confidence in managing symptoms and adhering to treatment protocols (Lorig and Holman, 2003). For patients with SLE, high self-efficacy serves as a psychological cornerstone. Patients with robust self-efficacy tend to exhibit greater proactivity when confronting pain and functional limitations, holding the conviction that their actions can positively contribute to health outcomes; this belief is strongly associated with lower depression levels and better social functioning (Rogers et al., 2005). Thus, we posit that self-efficacy provides the initial psychological impetus for identifying the positive aspects of the disease experience (Krok and Zarzycka, 2020).

However, the translation of self-efficacy into benefit finding is likely mediated by deeper self-regulatory processes, principally self-esteem. Self-esteem, the evaluative component of the self-concept, reflects an individual's overall sense of self-worth and acceptance (Rosenberg, 1965b). Chronic illness often acts as a threat to the self, leading to a “loss of self” and diminished worth (Charmaz, 1983). Self-efficacy acts as a protective buffer; when patients believe they can effectively manage their condition, this mastery experience reinforces their self-worth, thereby maintaining or elevating self-esteem (Mann et al., 2004). Individuals with high self-esteem possess greater psychological resources to buffer against stress and are more inclined to view the disease challenge as manageable rather than overwhelming (Creswell et al., 2005). Therefore, self-efficacy may first bolster self-esteem, providing the emotional stability required for subsequent adaptation.

While self-esteem provides the necessary psychological foundation, navigating the complexities of SLE requires concrete cognitive competencies, specifically health literacy. Health literacy is defined as the capacity to obtain, process, and understand basic health information needed to make appropriate health decisions (Sorensen et al., 2012). Patients with high self-esteem are more motivated to engage in health-promoting behaviors and invest in their own wellbeing (Ross et al., 2019). They are less likely to be paralyzed by fear and more likely to actively seek information, communicate with healthcare providers, and acquire disease management skills, thereby enhancing their health literacy (Schulz and Nakamoto, 2013; Xu et al., 2018). In turn, adequate health literacy empowers patients by reducing uncertainty. This cognitive clarity allows patients to reframe their illness experience from a chaotic disaster to a coherent narrative where growth is possible, directly facilitating benefit finding (Osborn et al., 2011). Conversely, low health literacy remains a barrier that impedes the cognitive processing necessary for finding meaning (Berkman et al., 2011; Baker et al., 2002).

Prior empirical inquiries have extensively documented the independent predictive value of these variables. For instance, the positive correlation between self-efficacy and benefit finding has been well-established in populations dealing with cancer and chronic pain (von Rezori et al., 2022; Cao et al., 2022). Moreover, self-esteem and health literacy have been identified as crucial intermediaries in psychological adaptation mechanisms in other clinical contexts. Research has demonstrated that self-esteem mediates the relationship between social support and life satisfaction (Cao and Liang, 2020), while health literacy has been shown to mediate the link between self-efficacy and health behaviors in patients with heart failure and diabetes (Chen et al., 2014; Lee Y. J. et al., 2016). However, the existing body of literature remains fragmented. Most studies have examined these variables in isolation or as simple parallel mediators, failing to elucidate the complex serial interplay, particularly within the specific context of SLE. To date, no study has systematically investigated the pathway from self-efficacy to benefit finding via the sequential influence of self-esteem and health literacy.

**Figure 1:**
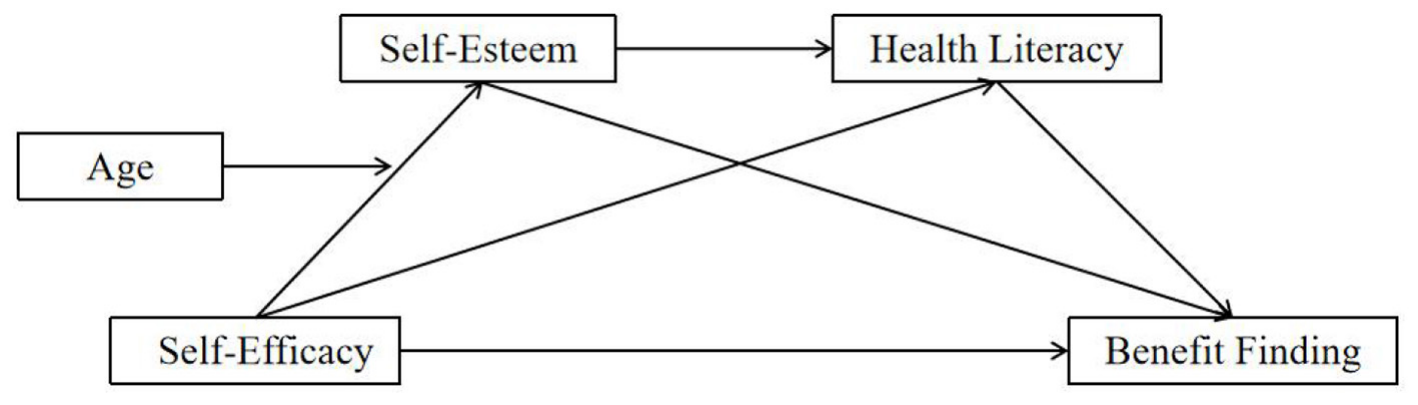
The hypothesized moderated serial mediation model.

To address this gap and provide a theoretical scaffold for these interactions, we draw upon Cognitive Adaptation Theory, which posits that adjustment to threatening events revolves around three themes: a search for meaning (benefit finding), an attempt to gain mastery (self-efficacy), and the process of self-enhancement (self-esteem) (Taylor, 1983; Park and Helgeson, 2006). Integrating this with the functional role of health literacy, we propose a specific serial mediation pathway. We hypothesize that self-efficacy enhances self-esteem, which in turn fosters proactive engagement in learning (health literacy), ultimately leading to the construction of positive meaning. Therefore, the aim of this study was to investigate the status of benefit finding among Chinese patients with SLE and to test the following hypotheses: (1) self-efficacy, self-esteem, health literacy, and benefit finding are positively correlated with each other; (2) self-esteem mediates the association between self-efficacy and benefit finding; (3) health literacy mediates the association between self-efficacy and benefit finding; (4) self-esteem and health literacy play a serial mediating role in the association between self-efficacy and benefit finding; and (5) Age moderates the relationship between self-efficacy and self-esteem. The proposed serial mediation model is illustrated in Figure 1.

## 2 Methods

### 2.1 Study design and participants

A cross-sectional survey design was employed for this study. Convenience sampling was utilized to recruit patients diagnosed with Systemic Lupus Erythematosus (SLE) from the outpatient department of a Grade 3A hospital in Zhejiang Province, China. The data collection period spanned from December 2022 to December 2024.

The inclusion criteria for participants were as follows: (1) Meeting the diagnostic criteria for SLE established by the American College of Rheumatology (ACR) in 1997 or the Systemic Lupus International Collaborating Clinics (SLICC) classification criteria in 2012; (2) Age ≥ 18 years; (3) Conscious with normal cognitive function and the ability to complete the questionnaire independently or with assistance; and (4) Willingness to participate in the study and provision of informed consent. Patients with severe organ failure, serious mental disorders, or other severe comorbidities were excluded.

The sample size was determined a priori using G^*^Power 3.1 software. We based our calculation on a Linear Multiple Regression model, which corresponds to the final step of the serial mediation analysis. The parameters were set as follows: a medium effect size (*f*^2^= 0.15), a significance level (α) of 0.05, and a high statistical power (1–β) of 0.95.To ensure the stability of the model estimates, we adopted a conservative approach by accounting for 13 predictors, which includes the main study variables (self-efficacy, self-esteem, health literacy) and potential demographic covariates. The calculation indicated a minimum required sample size of 189. Our study included 215 valid participants, which exceeds this requirement. This affirms that the current sample size is sufficient to ensure high statistical power and the stability of the serial mediation results.

### 2.2 Instruments

#### 2.2.1 Sociodemographic and clinical questionnaire

A self-designed questionnaire was developed to collect baseline characteristics. Variables were selected based on their potential influence on disease burden, economic status, health literacy, factors: (1) Gender, age, marital status, and religious beliefs. (2) Socioeconomic status: educational level, occupational status, monthly income, and method of medical payment. (3) Clinical characteristics: disease duration and comorbidities (presence of diseases other than SLE).

#### 2.2.2 Measurement of self-efficacy

Self-efficacy was assessed using the General Self-Efficacy Scale (GSES), originally developed by (Zhang and Schwarzer 1995) and validated in Chinese by (Wang et al. 2001). This 10-item instrument measures an individual's confidence in coping with daily challenges and stressful situations. Items are rated on a 4-point Likert scale ranging from 1 (“not at all true”) to 4 (“exactly true”). The total score is calculated by summing the item scores, with higher scores indicating a higher level of self-efficacy. In the present study, the Cronbach's alpha coefficient for the scale was 0.843.

#### 2.2.3 Measurement of self-esteem

Self-esteem was evaluated using the Chinese version of the Rosenberg Self-Esteem Scale (RSES). Originally developed by (Rosenberg 1965a) and validated for the Chinese population by (Dai 2015), this 10-item instrument assesses an individual's global sense of self-worth and self-acceptance. Participants rated each item on a 4-point Likert scale ranging from 1 (“strongly agree”) to 4 (“strongly disagree”). To ensure consistency, items 3, 5, 8, 9, and 10 were reverse-scored. Consequently, total scores range from 10 to 40, with higher aggregate scores indicating higher levels of self-esteem. The Chinese version of the RSES has demonstrated good psychometric properties in previous research, with a reported test-retest reliability of 0.72 and a Cronbach's α coefficient of 0.88. In this study, the Cronbach's alpha coefficient for the Rosenberg Self-Esteem Scale was 0.854.

#### 2.2.4 Measurement of health literacy

Health literacy was evaluated using the Health Literacy Scale for Chronic Patients (HLSCP), developed by (Sun 2012). This instrument assesses four key dimensions: information acquisition ability, communicative interaction, willingness to improve health, and willingness to seek economic support. The scale comprises 24 items rated on a 5-point Likert scale ranging from 1 (“no difficulty”) to 5 (“very difficult”). All items were reverse-coded so that higher total scores reflected higher levels of health literacy. In this study, the scale demonstrated excellent internal consistency (Cronbach's α = 0.894).

#### 2.2.5 Measurement of benefit finding

Benefit finding was assessed using the Benefit Finding Scale (BFS), originally adapted by (Weaver et al. 2008) and subsequently revised for Chinese populations by (Liu et al. 2014). This 22-item instrument measures positive changes following illness across six dimensions: acceptance, family relations, worldview, personal growth, social relations, and health behaviors. Items are rated on a 5-point Likert scale ranging from 1 (“not at all”) to 5 (“extremely”). The total score is calculated by summing the item scores, with higher scores indicating a greater perception of benefits derived from the illness experience. In the current study, the scale demonstrated excellent internal consistency (Cronbach's α = 0.950).

### 2.3 Data collection

To ensure the quality of data collection, the survey was conducted by three researchers with medical backgrounds who underwent standardized training. The training covered the study purpose, questionnaire content, communication skills, and ethical considerations. The investigators explained the purpose and significance of the study to the participants, obtained their informed consent, and provided necessary assistance to those who had difficulty reading or writing. All questionnaires were checked for completeness on-site, and any missing items were immediately addressed.

### 2.4 Statistical analysis

Statistical analyses were performed using IBM SPSS Statistics 25.0. Given the use of self-reported measures, Harman's single-factor test was conducted to assess potential common method bias. Descriptive statistics were utilized to summarize the demographic and clinical characteristics of the participants; continuous variables were expressed as mean ± standard deviation (SD), while categorical variables were presented as frequencies (n) and percentages (%). Pearson correlation analysis was conducted to examine the bivariate associations among self-efficacy, self-esteem, health literacy, and benefit finding. To test the hypothesized serial mediation model, the PROCESS macro (Model 6) developed by Hayes was employed. The significance of the indirect effects was evaluated using 5,000 bootstrap resamples to generate bias-corrected 95% confidence intervals (CI). An indirect effect was considered statistically significant if the 95% CI did not contain zero. All statistical tests were two-tailed, with the significance level set at *P* < 0.05.

### 2.5 Ethical approval

Ethical approval for this study was obtained from the Institutional Review Board of Zhejiang Shuren University (Approval No. 20200105). Participation was strictly voluntary and anonymous, and informed consent was obtained from all participants prior to data collection. Participation was voluntary, anonymity was guaranteed.

## 3 Results

### 3.1 Assessment of common method bias

Since the data were collected using self-report questionnaires, common method bias (CMB) was a potential concern. Additionally, given the cross-sectional design, it is important to clarify that terms such as “influence,” “predict,” and “effect” used in the following results refer to statistical dependence and variance explanation derived from the regression and mediation analyses. These terms are used in their statistical sense and do not imply strictly proven causal relationships or temporal sequences among the variables. We conducted Harman's single-factor test to assess the severity of CMB. All measurement items were entered into an unrotated exploratory factor analysis. The results showed that 13 factors with eigenvalues greater than 1 were extracted. The first factor explained 27.86% of the total variance, which is well below the critical threshold of 40% (Xiong et al., 2012). These results indicate that common method bias is not a serious issue in the present study.

### 3.2 Demographic and clinical characteristics

A total of 215 paper questionnaires were distributed on-site at a tertiary hospital. After excluding incomplete responses, 207 valid questionnaires were included in the final analysis, yielding an effective response rate of 96.28%. The detailed characteristics of the study population are presented in Table 1. The participants were predominantly female (94.20%), with the majority falling within the 18–39 age group. Regarding socioeconomic status, the largest proportion (45.41%) had an educational level of high school or vocational college. More than half of the participants were currently employed, and only a small minority (5.80%) reported a monthly income of less than 3,000 RMB. In terms of social and spiritual support, 55.07% were married, while the vast majority (91.85%) reported no religious affiliation; among those who did (8.15%), Buddhism was the most common. Clinically, nearly half of the patients (47.34%) reported no comorbidities other than SLE. The disease duration for most participants was concentrated in the range of 0.5 to 10 years. Regarding medical expense coverage, the majority of participants (58.46%) utilized medical insurance.

**Table 1:** Socio-demographic and clinical characteristics of the participants (*n* = 207).

**Variables**	**Characteristics**	***n***	**%**
Gender	Female	195	94.20
	Male	12	5.80
Education level	Junior high school or below	37	17.87
	High school/Junior college	94	45.41
	Bachelor's degree or above	76	36.71
Age (years)	18–29	42	20.29
	30–39	68	32.85
	40–49	61	29.47
	≥50	36	17.39
Monthly income (CNY)	< 3,000	12	5.80
	3,000–7,000	82	39.61
	7,000–11,000	94	45.41
	>11,000	19	9.18
Religious belief	Yes	21	10.14
	No	186	89.86
Marital status	Married	114	55.07
	Unmarried	83	40.10
	Other	10	4.83
Employment status	Self-employed	47	22.70
	Employed	119	57.49
	Other	41	19.81
Presence of comorbidities	No	98	47.34
	Yes	109	52.66
Disease duration (years)	0.5–5	93	44.93
	6–10	53	25.60
	11–15	24	11.59
	16–20	19	9.18
	>20	18	8.70
Medical payment method	Out-of-pocket	14	6.76
	Medical insurance	121	58.46
	NRCMS	72	34.78

NRCMS, New Rural Cooperative Medical Scheme; CNY, Chinese Yuan.

### 3.3 Descriptive statistics of study variables

As shown in Table 2, the mean scores for self-efficacy and self-esteem were 21.47 ± 5.37 and 27.42 ± 2.68, respectively. The participants reported an average health literacy score of 94.96 ± 11.42. Furthermore, the mean level of benefit finding within this cohort was 65.20 ± 10.92. Pearson correlation analysis indicated that self-efficacy, self-esteem, health literacy, and benefit finding were significantly correlated with each other (Table 3).

**Table 2:** Current status of self-efficacy, self-esteem, health literacy, and perceived benefits of illness in patients with SLE.

**Variables**	**Dimensions**	**Total score (Mean ±SD)**	**Item mean score (Mean ±SD)**
Self-efficacy	Total score	21.47 ± 5.37	2.15 ± 0.54
Self-esteem	Total score	27.42 ± 2.68	2.74 ± 0.27
Health literacy	Total score	94.29 ± 10.71	3.93 ± 0.45
	Information acquisition	37.58 ± 5.67	4.18 ± 0.63
	Communication interaction	31.60 ± 5.96	3.51 ± 0.66
	Health improvement	16.62 ± 2.53	4.15 ± 0.63
	Economic support	8.49 ± 1.68	4.25 ± 0.84
Benefit finding	Total score	65.20 ± 10.92	2.96 ± 0.50
	Acceptance	14.14 ± 3.80	3.53 ± 0.95
	Family relations	8.94 ± 2.41	2.36 ± 0.60
	Worldview	7.38 ± 3.61	2.24 ± 0.60
	Personal growth	16.35 ± 2.75	4.09 ± 0.69
	Social relations	9.45 ± 2.40	3.15 ± 0.80
	Health behaviors	8.94 ± 2.36	2.98 ± 0.79

**Table 3:** Correlations among self-efficacy, self-esteem, health literacy, and benefit finding in patients with SLE.

**Variables **	**Self-efficacy **	**Self-esteem **	**Health literacy **	**Benefit finding **	**Information acquisition **	**Communicationinteraction **	**Health improvement **	**Economic support **	**Acceptance **	**Family relation **	**Worldview **	**Personal growth **	**Social relations **	**Health behaviors **
Self-efficacy	1.00													
Self-esteem	0.842^***^	1.00												
Health literacy	0.885^***^	0.836^***^	1.00											
Benefit finding	0.833^***^	0.846^***^	0.858^***^	1.00										
Information acquisition	0.504^***^	0.482^***^	0.693^***^	0.513^***^	1.00									
Communication interaction	0.745^***^	0.713^***^	0.760^***^	0.741^***^	0.157^*^	1.00								
Health improvement	0.564^***^	0.511^***^	0.564^***^	0.475^***^	0.125	0.385^***^	1.00							
Economic support	0.456^***^	0.410^***^	0.496^***^	0.398^***^	0.303^***^	0.190^***^	0.310^***^	1.00						
Acceptance	0.460^***^	0.488^***^	0.455^***^	0.631^***^	0.335^***^	0.404^***^	0.180^***^	0.071	1.00					
Family relation	0.499^***^	0.503^***^	0.448^***^	0.478^***^	0.232^***^	0.385^***^	0.290^***^	0.276^***^	0.031	1.00				
Worldview	0.593^***^	0.557^***^	0.588^***^	0.736^***^	0.305^***^	0.553^***^	0.328^***^	0.266^***^	0.262^***^	0.401^***^	1.00			
Personal growth	0.357^***^	0.462^***^	0.469^***^	0.524^***^	0.339^***^	0.362^***^	0.248^***^	0.186^***^	0.219^***^	0.067	0.228^***^	1.00		
Social relations	0.639^***^	0.595^***^	0.673^***^	0.683^***^	0.372^***^	0.562^***^	0.412^***^	0.423^***^	0.298^***^	0.210^***^	0.361^***^	0.283^***^	1.00	
Health behaviors	0.634^***^	0.621^***^	0.650^***^	0.693^***^	0.358^***^	0.546^***^	0.406^***^	0.389^***^	0.323^***^	0.233^***^	0.411^***^	0.206^***^	0.572^***^	1.00

Significance levels: ^***^P < 0.001, ^*^P < 0.05.

### 3.4 Multicollinearity diagnostics

Given the strong correlations observed among the independent variables (*r* > 0.8), we assessed the potential risk of multicollinearity by examining the Variance Inflation Factor (VIF) and tolerance statistics. The results showed that the VIF values were 5.50 for self-efficacy, 3.95 for self-esteem, and 5.31 for health literacy. Although these values indicate some degree of shared variance, they are all below the widely accepted threshold of 10 (Hair et al., 2010; Neter et al., 1996), suggesting that multicollinearity is not severe enough to distort the parameter estimates. Correspondingly, the tolerance values ranged from 0.18 to 0.25, all exceeding the critical cutoff of 0.10. These diagnostics confirm that the regression model is statistically robust.

### 3.5 Mediating effect test

**Figure 2:**
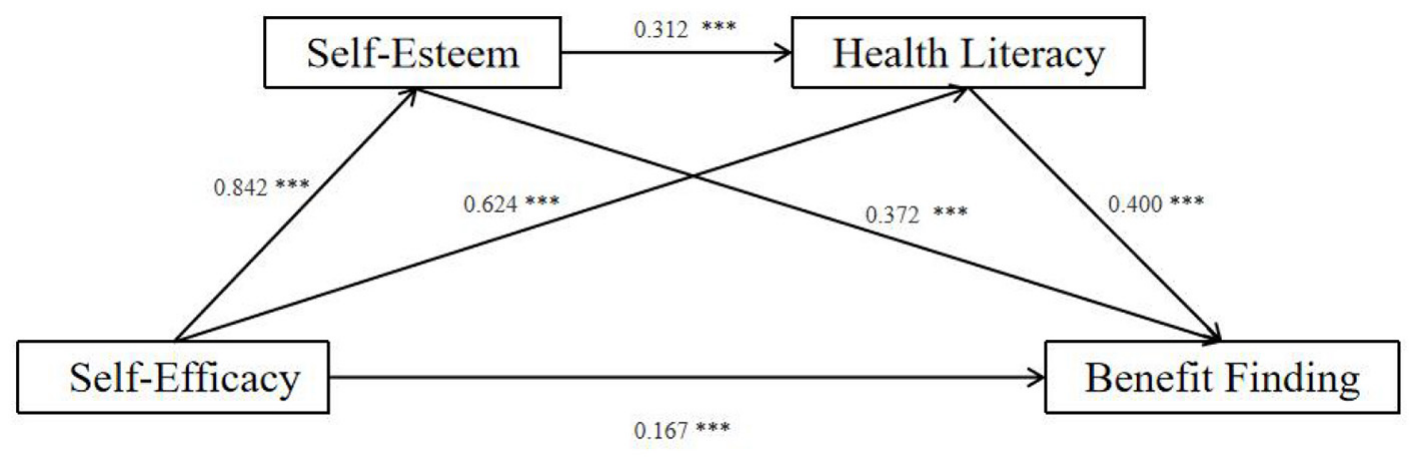
The serial mediation model illustrating the relationships among self-efficacy, self-esteem, health literacy, and benefit finding. ***P < 0.001.

The serial mediation analysis was conducted using the PROCESS macro (Model 6) to investigate the mediating roles of self-esteem and health literacy in the relationship between self-efficacy and benefit finding. As shown in Table 4 and Figure 2, the regression model for self-esteem (Model 1) was statistically significant (*F* = 499.30, *P* < 0.001), accounting for 70.9% of the variance (*R*^2^= 0.709). Specifically, self-efficacy significantly and positively associated with self-esteem (*B* = 0.420, *P* < 0.001). For health literacy (Model 2), the model showed a good fit (*F* = 439.77, *P* < 0.001) and explained 81.2% of the total variance (*R*^2^= 0.812). The results indicated that both self-efficacy (*B* = 1.243, *P* < 0.001) and self-esteem (*B* = 1.242, *P* < 0.001) were significant positive correlates. Regarding the outcome variable, benefit finding (Model 3), the regression model was also significant (*F* = 264.02, *P* < 0.001), explaining 79.6% of the variance (*R*^2^= 0.796). Self-efficacy (*B* = 0.339, *P* < 0.05), self-esteem (*B* = 1.513, *P* < 0.001), and health literacy (*B* = 0.407, *P* < 0.001) all showed significant positive associations with benefit finding.

**Table 4:** Regression analysis of the relationship between variables in the chain mediation model.

**Model**	**Outcome**	**Predictor**	***R***	***R^2^***	***F***	***B***	***t***	**LLCI**	**ULCI**
Model 1	Self-esteem	SE	0.842	0.709	499.299^***^	0.420	22.345	0.383	0.457
Model 2	HL	SE	0.901	0.812	439.767^***^	1.243	11.074	1.022	1.465
		Self-esteem				1.242	5.518	0.798	1.686
Model 3	BF	SE	0.892	0.796	264.023^***^	0.339	2.241	0.041	0.636
		Self-esteem				1.513	5.899	1.008	2.019
		HL				0.407	5.471	0.261	0.554

SE, Self-Efficacy (in pathways) or Standard Error (in column headers); BF, Benefit Finding; HL, Health Literacy. Significance levels: ^***^P < 0.001.

The bootstrap results (Table 5) revealed a significant total effect of self-efficacy on benefit finding (β = 1.693, SE = 0.078, 95% CI: 1.538, 1.848). After controlling for the mediators, the direct effect remained significant (β = 0.339, SE = 0.151, 95% CI: 0.041, 0.636), indicating that self-esteem and health literacy played a partial mediating role. This direct path accounted for 20.02% of the total effect. The total indirect effect was 1.354 (SE = 0.142, 95% CI: 1.095, 1.655), which constituted 79.98% of the total effect. Specifically, the analysis confirmed three significant indirect pathways, as the 95% confidence intervals for all paths did not encompass zero: Ind1 (via Self-esteem): Self-efficacy → Self-esteem → Benefit Finding. This was the strongest mediating path (β = 0.636, 95% CI: 0.234, 1.067), accounting for 37.57% of the total effect. Ind2 (via Health Literacy): Self-efficacy → Health Literacy → Benefit Finding. This path was significant (β = 0.506, 95% CI: 0.200, 0.819), accounting for 29.89% of the total effect. Ind3 (Serial Mediation): Self-efficacy → Self-esteem → Health Literacy → Benefit Finding. The serial mediating pathway was also significant (β = 0.212, 95% CI: 0.042, 0.577), accounting for 12.52% of the total effect.

**Table 5:** Total, direct, and indirect effects of the serial mediation model.

**Pathways**		**Effect**	**SE**	**95%CI**		**Relative effect (%)**
				**LL**	**UL**	
Total effect		1.693	0.078	1.538	1.848	
Direct effect		0.339	0.151	0.041	0.636	20.02
Total indirect effect(s)		1.354	0.142	1.095	1.655	79.98
Ind1	SE → Self-Esteem → BF	0.636	0.217	0.234	1.067	37.57
Ind2	SE → HL → BF	0.506	0.157	0.200	0.819	29.89
Ind3	SE → Self-Esteem → HL → BF	0.212	0.141	0.042	0.577	12.52

SE, Self-Efficacy (in pathways) or Standard Error (in column headers); BF, Benefit Finding; HL, Health Literacy; CI, Confidence Interval; LL, Lower Limit; UL, Upper Limit; Ind, Indirect effect.

### 3.6 Test for the moderating effect of age in the chain mediation model

**Figure 3:**
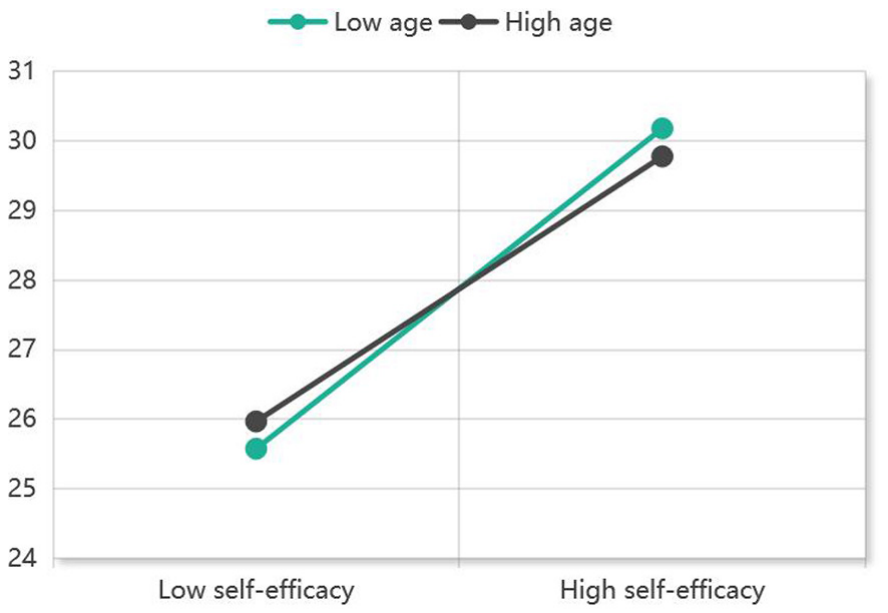
Simple slope plot of the moderating effect of age on the relationship between self-efficacy and self-esteem.

Regression analysis was performed using Model 83 of the PROCESS macro. After incorporating age into the chain mediation model, the interaction term between self-efficacy and age showed a significant negative correlation with self-esteem (β= −0.080, *t* = −3.837, *P* < 0.001). This indicates that age moderates the effect of self-efficacy on self-esteem within the pathway of “self-efficacy → self-esteem → health literacy → benefit finding” (Table 6). To further interpret the nature of this moderating effect, a simple slope analysis was conducted (Figure 3). The results showed that at low levels of age (−0.203), self-efficacy had a strong positive association with self-esteem (β = 0.461, *P* < 0.001, 95% CI: 0.411–0.511). Conversely, at high levels of age (0.797), the positive association between self-efficacy and self-esteem was relatively weaker (β = 0.381, *P* < 0.001, 95% CI: 0.327–0.436). The index of moderated mediation confirmed significance, with a 95% confidence interval of [−0.121, −0.005], which excluded zero. In conclusion, the lower the age, the stronger the indirect effect of self-efficacy on benefit finding through the chain mediating role of self-esteem and health literacy.

**Table 6:** The moderating effect of age on the relationship between self-efficacy and self-esteem.

**Dependent variable**	**Independent variable**	**β**	**SE**	***t***	***P***	**95% CI**
Self-esteem	Self-efficacy	0.445	0.025	18.094	< 0.001	0.396 to 0.493
	Age	0.038	0.167	0.225	0.822	−0.292 to 0.367
	Self-efficacy × Age	−0.080	0.021	−3.837	< 0.001	−0.120 to −0.039

## 4 Discussion

### 4.1 Status of self-efficacy, self-esteem, health literacy, and benefit finding in patients with SLE

The present study revealed that patients with Systemic Lupus Erythematosus exhibited moderate levels of benefit finding. This finding aligns with recent research by (Yang et al. 2025), suggesting that despite the chronic and unpredictable nature of the disease, patients possess the psychological resilience to perceive positive life changes, such as personal growth and deepened relationships. However, the scores observed were higher than those reported in certain cancer populations (He et al., 2025; Liu et al., 2025). This interesting discrepancy may be attributed to the protracted, lifelong nature of Systemic Lupus Erythematosus. Unlike the sudden, acute, and often life-threatening crisis associated with a cancer diagnosis, SLE typically follows a chronic trajectory spanning decades. This extended duration may afford patients a longer timeframe to cognitively process their illness experience, mobilize adaptive coping strategies, and gradually integrate the condition into their identity. Consequently, this prolonged process of adaptation may foster a more profound and sustained sense of personal growth and benefit finding compared to the acute distress phase often captured in cancer studies (Lei et al., 2025). Regarding the predictor variables, participants reported suboptimal to moderate levels of health literacy and self-efficacy. This echoes the findings of (Sheehan et al. 2025) and (Zhang et al. 2025), indicating that the complexity of lifelong treatment regimens and the lack of tailored educational resources often leave patients feeling ill-equipped to manage their condition. Limited health literacy is associated with patients' comprehension of complex treatments, which in turn erodes their self-efficacy. This fosters a vicious cycle of disempowerment, trapping patients in a passive role and impeding their ability to become active partners in their own care.

Furthermore, self-esteem levels were variable, consistent with previous studies (Thapa et al., 2025; Ferreira Maciel et al., 2026). This likely reflects the potential association with visible symptoms, such as malar rash and alopecia, as well as corticosteroid-induced body image changes, which are well-documented factors that may diminish self-worth in this population (Hale et al., 2006). Additionally, the prevalence of comorbid anxiety and depression in this population remains high, further complicating the psychological landscape (Caruso Mazzolani et al., 2023).

### 4.2 Correlation analysis of study variables

The correlation analysis provided detailed insights into the interrelationships among the study variables, all of which were statistically significant and positive. First, a strong positive correlation was observed between self-efficacy and self-esteem. This supports the theoretical proposition by (Bandura 1997) that efficacy beliefs are a primary source of self-worth. When patients successfully manage daily symptoms or adhere to complex medication regimens, their sense of competence is reinforced, which in turn bolsters their overall self-esteem (Ma et al., 2025). Second, self-efficacy was positively associated with health literacy. This suggests that confidence serves as a motivational catalyst; patients who believe in their ability to control their health are more prone to actively seek, process, and utilize health information (Chen et al., 2025; Wang et al., 2025). Third, a positive relationship was found between self-esteem and health literacy. This association implies that self-worth is linked to health behaviors (Li, 2024). Patients who value themselves highly are more likely to view their health as a worthy investment, thereby demonstrating greater engagement in acquiring the literacy skills necessary for self-care (Jafarigiv and Peyman, 2019). Finally, all three independent variables—self-efficacy, self-esteem, and health literacy—were positively correlated with benefit finding. This consistency underscores that psychological adaptation in chronic illness is not driven by a single factor but is the result of a cumulative interplay of cognitive confidence, emotional self-regard, and functional health competence (Maras et al., 2022).

### 4.3 The direct effect of self-efficacy on benefit finding

Consistent with the initial hypothesis, self-efficacy was identified as a significant direct correlate to benefit finding. Self-efficacy serves as a motivational foundation for coping. Patients with a high sense of self-efficacy view disease-related challenges as manageable tasks rather than insurmountable threats (Bandura, 1997). This sense of agency empowers them to adopt active coping strategies, such as cognitive reframing and seeking social support, rather than resorting to avoidance or resignation (Napolitano et al., 2025). By maintaining assurance in their ability to control symptoms, patients can reduce psychological distress and create a mental environment conducive to post-traumatic growth and finding meaning in adversity (Kim et al., 2024; Owens, 2016).

### 4.4 The mediating role of self-esteem

A key finding of this study is the mediating role of self-esteem between self-efficacy and benefit finding. This perceived agency counteracts the feelings of helplessness and body dissatisfaction often induced by the disease, thereby preserving global self-worth (Mikula et al., 2018). Subsequently, elevated self-esteem serves as a vital psychological buffer. It mitigates the threat appraisal associated with chronic illness, allowing patients to transcend a victim identity (Peyser et al., 2024). With a fortified sense of self, patients possess the cognitive flexibility to reframe their adverse experiences, shifting focus from physical limitations to personal growth and resilience (Cho et al., 2025; Maurin and Martinent, 2023). Thus, self-esteem functions as an affective bridge, converting the behavioral confidence of self-efficacy into the cognitive restructuring necessary for benefit finding (Southward et al., 2024; Kam et al., 2024).

### 4.5 The mediating role of health literacy

Health literacy was also confirmed as a significant mediator. Instead of merely representing the accumulation of disease-related knowledge, health literacy reflects the functional capacity to acquire and utilize information (Ye et al., 2025; Liu et al., 2024). Patients with high self-efficacy are more likely to mobilize their health literacy skills, such as actively communicating with healthcare providers and engaging in information acquisition (Chen et al., 2025). Rather than implying a simple increase in knowledge, high self-efficacy motivates patients to utilize their ability to seek economic support and improve their health status. When patients effectively exercise these health literacy competencies, they can reduce the uncertainty and complexity associated with SLE (Li, 2024; Wang et al., 2024). This capacity to obtain and process necessary information relieves cognitive burden, allowing patients to redirect their mental energy from chaos to identifying positive life changes or benefit finding (Delis, 2019; Warchoł-Biedermann et al., 2022).

### 4.6 The serial mediating effect

Most importantly, this study confirmed the serial mediation pathway linking self-efficacy, self-esteem, health literacy, and benefit finding. This chain reaction reveals a sequential mechanism moving from psychological beliefs to self-evaluation, and finally to cognitive capability. Specifically, self-efficacy is associated with enhanced self-esteem by validating the capability of the patient. Individuals with higher self-worth are, in turn, more motivated to invest in their own wellbeing (Chen et al., 2025; Lu et al., 2022). This motivation is reflected in a greater willingness to employ health literacy skills, such as information acquisition and communicative interaction. Consequently, the effective use of these literacy skills empowers patients to navigate the complexities of their illness trajectory (Nakayama et al., 2022). The combination of psychological resilience and the active application of cognitive competence creates a synergistic effect, associated with the patient's ability to thrive and discover profound meaning in their journey (Bartrés-Faz et al., 2018; Du et al., 2025; Ozonder Unal and Ordu, 2023). It is important to note that, given the cross-sectional nature of this study, these findings reflect positive associations among patients with adequate health literacy, rather than confirming that intervention in one variable strictly causes improvement in the subsequent variables.

### 4.7 Negative moderating effect of age on the relationship between self-efficacy and self-esteem

The results of this study indicated that age plays a negative moderating role in the relationship between self-efficacy and self-esteem among patients with SLE. specifically, the positive association between self-efficacy and self-esteem diminishes with increasing age. Simple slope analysis further confirmed that the impact of self-efficacy on self-esteem was more pronounced in younger patients with SLE. This suggests that the pathway by which self-efficacy contributes to benefit finding via self-esteem is particularly critical in the younger population. This finding may be attributed to the distinct psychological developmental characteristics and social role tasks associated with different life stages, aligning with the Life Cycle Theory (Hussain et al., 2022). Young and middle-aged patients with SLE are in a critical period of physical and mental development, facing challenges from multiple social roles, including academic pursuits, career competition, marriage, and child-rearing (Shi et al., 2022). High self-efficacy signifies a strong ability to cope with disease interference and maintain social functioning. This ability is crucial for preserving the sense of self-worth in young patients, thereby significantly enhancing their self-esteem (Nasiriziba et al., 2020). In contrast, with advancing age, individual self-concepts and values tend to become more mature and stable (Orth et al., 2018). Older patients derive self-esteem from broader and more diverse sources, relying less on specific physical functions or immediate coping abilities. The elderly generally have psychological expectations regarding physiological decline; thus, even if self-efficacy fluctuates, its relationship with self-esteem remains relatively limited. Based on these findings, it is recommended that healthcare providers adopt stratified intervention strategies in clinical practice. Young patients with SLE should be prioritized for psychological interventions, focusing on enhancing their self-efficacy through methods such as Cognitive Behavioral Therapy (CBT) and peer support programs. For older patients, while improving self-efficacy, attention should also be paid to leveraging their past life experiences and family support systems to maintain self-esteem from multiple dimensions, thereby promoting psychological wellbeing and benefit finding.

### 4.8 Clinical implications

Consequently, clinical interventions should transition from passive health education and simple information dissemination to comprehensive skill-building programs. Healthcare providers should focus on empowering patients with the practical tools necessary to navigate the healthcare system effectively. This includes training patients on how to identify and access high-quality health resources, how to interpret clinical information or treatment regimens, and how to apply this knowledge to their daily self-management and decision-making processes. By fostering these core competencies of health literacy alongside self-efficacy and self-esteem, clinicians can more effectively support patients in their journey toward benefit finding and psychological resilience.

### 4.9 Limitations and future directions

Several limitations of the present study should be acknowledged. First and foremost, the cross-sectional design limits our ability to make strict causal inferences. Although the hypothesized serial mediation model is grounded in Social Cognitive Theory and previous literature, the data were collected at a single time point. Consequently, we cannot empirically confirm the temporal sequence of these variables, nor can we rule out the possibility of bidirectional relationships or reverse causality. Second, the reliance on self-reported measures represents a notable limitation. Although validated scales were used, subjective responses may be influenced by social desirability bias, recall bias, or the patients' immediate emotional state, potentially affecting the accuracy of the data. Third, the generalizability of the findings is limited by the sampling method and study population. Participants were recruited from the outpatient department of a single tertiary hospital in Zhejiang Province via convenience sampling, which may not represent patients with SLE in other regions or community settings. Additionally, the sample was predominantly female (94.20%); while this reflects the known epidemiological prevalence of SLE, caution should be exercised when generalizing these results to male patients. Furthermore, as the study excluded in patients with severe organ failure or acute complications, the findings may not accurately reflect the benefit finding and psychological mechanisms of patients in critical or unstable conditions. Finally, this study primarily focused on intra-personal psychological resources such as self-efficacy; therefore, external environmental factors, including family support and economic pressure, were not included in the current model.

Based on these limitations, several directions for future research are proposed. First, to address the issue of causality, longitudinal designs should be adopted to track the dynamic changes of these variables over time and verify the proposed relationships. Second, to enhance generalizability, future studies should recruit broader and more representative samples—including patients from different geographical regions, varying disease severities, and a higher proportion of males. Third, to mitigate potential self-report bias, researchers should combine subjective measures with objective indicators (e.g., health knowledge tests, medical record compliance, or the SLEDAI index) or peer assessment tools. Lastly, future research should expand the theoretical framework by incorporating both internal and external factors (such as family support and economic status) to build a more comprehensive, multidimensional model.

## 5 Conclusion

In conclusion, this study elucidates the complex pathways linking self-efficacy to benefit finding in patients with Systemic Lupus Erythematosus. Self-efficacy is positively associated with benefit finding directly and indirectly through the independent and serial mediating roles of self-esteem and health literacy. These findings highlight the necessity of integrating psychological empowerment, self-worth enhancement, and health literacy training into routine care to facilitate positive adaptation.

## Data Availability

The raw data supporting the conclusions of this article will be made available by the authors, without undue reservation.
